# Impact of emotion regulation on emotional experiences following social rejection: an ERP study

**DOI:** 10.3389/fpsyg.2025.1575002

**Published:** 2025-08-29

**Authors:** Dengfeng Xie, Jiamei Lu, Zhangming Xie

**Affiliations:** ^1^School of Education and Continuing Education, West Anhui University, Lu’an, China; ^2^School of Psychology, Shanghai Normal University, Shanghai, China

**Keywords:** cognitive reappraisal, attention transfer, social rejection, emotion regulation, event-related potentials

## Abstract

Social rejection elicits potent emotional responses with significant mental health implications. This event-related potential (ERP) study characterized the neurochronometry of emotion regulation during social rejection. Key findings revealed that (1) linear mixed-effects modeling confirmed attention transfer significantly reduced late positive potential (LPP) amplitudes versus cognitive reappraisal and non-regulation across all time windows; (2) the superiority of attention transfer was most pronounced during the early/mid-processing stages, achieving rapid disengagement from rejection stimuli; (3) enhanced suppression under social rejection reflected context-dependent regulatory dynamics. Attention transfer demonstrates contextually optimized efficacy for rapid threat disengagement during social rejection. However, EEG source localization limitations preclude definitive conclusions regarding dorsolateral prefrontal cortex (DLPFC) engagement; future studies should employ multimodal approaches (e.g., fMRI-EEG) to verify neuroanatomical mechanisms.

## 1 Introduction

Social rejection is defined as exclusion from social groups or interactions and directly threatens fundamental needs for belonging and connection (Du and Xia, 2008; MacDonald and Leary, 2005). This deprivation triggers unmet relational needs and psychological strain, thereby escalating maladjustment risks (Kavakli, 2021; Prinstein and Giletta, 2016). Thus, effective regulation of socially derived negative emotions is a critical determinant of mental well-being (Stone, 2022).

Given this imperative, two dominant strategies have emerged as key research foci: cognitive reappraisal–an emotion regulation strategy involving stimulus reevaluation to alter emotions, comprising subtypes such as positive reappraisal (focusing on situational benefits/“looking on the bright side”) and detached reappraisal (adopting an objective perspective via psychological distancing; Ochsner et al., 2012)–and attention transfer (redirecting focus from emotional stimuli; Kozubal et al., 2023). Neurobehavioral evidence confirms both cognitive reappraisal and attention transfer demonstrate efficacy (Brockman et al., 2023; Specker et al., 2024), yet their neurophysiological profiles fundamentally diverge: (1) cognitive reappraisal subtypes exhibit nuanced dynamics wherein detached reappraisal attenuates negative emotions more effectively than positive reappraisal (which sustains positive affect; Denny and Ochsner, 2014; Shiota and Levenson, 2009), alongside inconsistent late positive potential (LPP) modulation (Moser et al., 2009; Webster et al., 2022); whereas (2) attention transfer suppresses amygdala activity (Kanske et al., 2012) yet paradoxically elevates LPP amplitudes while reducing subjective negativity (Wang et al., 2015)–a dissociation contradicting established emotion regulation physiology (Paul et al., 2016). Crucially, LPP segmentation into early (300–600 ms), mid-stage (600–1000 ms) and late (1000–1500 ms) windows follows standardized chronometric protocols for capturing stage-specific regulatory dynamics (Langeslag and van Strien, 2010). Subsequent research confirms that electrophysiological modulation precedes subjective effects (Wang et al., 2024).

These discrepancies highlight a critical neurochronometric gap: whether early-stage sensory gating during attention transfer enables more effective threat disengagement than cognitive reappraisal during acute social rejection. We thus propose the Threat-Adaptive Disengagement Model, positing that under high-threat conditions, early attentional filtering rapidly mobilizes dorsolateral prefrontal resources through working memory occupation (e.g., serial subtraction), blocking emotional stimuli from evaluative circuits. Alternatively, cognitive reconstruction relies on slower prefrontal reconfiguration, rendering it vulnerable to stress-induced resource depletion.

Based on this model and extant evidence, we hypothesize the following:

Early-stage (300–600 ms): Attention transfer will elicit significantly lower LPP amplitudes than both cognitive reappraisal and non-regulation, reflecting immediate sensory gating (H1).

Mid-stage (600–1000 ms): Attention transfer maintains superior LPP suppression through sustained resource mobilization, while cognitive reappraisal shows only partial efficacy (H2).

Late-stage (1000–1500 ms): Attention transfer sustains significant LPP reduction, whereas cognitive reappraisal achieves merely compensatory effects due to delayed prefrontal engagement (H3).

## 2 Methods

### 2.1 Participants

The sample size was determined based on an *a priori* power analysis, which indicated that 24 participants were required to achieve a test power of 0.80 (α = 0.05) at a medium effect size (0.25). Consequently, this study was adequately powered. The participants in this study comprised 27 college students (including 16 men and 11 women), whose ages ranged from 19 to 23 years, with a mean age of 21.14 years and a standard deviation of 1.54 years, allowing 11% redundancy. During the data preprocessing stage, the EEG data quality from two college students did not meet the established standards owing to numerous artifacts and other adverse factors. Consequently, data from two participants were excluded. The final analysis included 25 effective subjects (14 men, 11 women), exceeding the power threshold. Gender distribution reflected the volunteer pool but was not analyzed separately due to limited cell sizes. All subjects involved in the experiment were right-handed, had normal or corrected to normal vision, confirmed that they had no history of brain injury and did not report any form of mental disorder. Before the experiment, all subjects signed the informed consent. The participants were explicitly informed about potential exposure to socially themed images that might elicit discomfort during the experimental procedure. Experimental sessions were immediately terminated upon participant request. All individuals received counseling service referrals post-experimentation, with systematic 48-h follow-up contacts confirming the absence of persistent adverse reactions.

### 2.2 Experimental materials

Visual stimuli depicting social situations were standardized for size, luminance, and viewing angle (10° horizontal visual angle) using Adobe Photoshop CS6. The fixation point “+” was located in the center of the screen, and its horizontal and vertical angles were both 0.5°, while the presentation of the guidance language occupied 4.5° and 1.3° of horizontal and vertical angles, respectively.

The remarkable effect of social situation pictures on emotion induction has become a widely used stimulus material in the field of EEG experiments (Cano et al., 2009). A total of 520 picture materials were collected. Subsequently, the 40 college students were invited to score the pictures on a 9-point emotional scale. Based on the scores for social rejection, two types of emotional pictures depicting social situations were ultimately selected, with 81 pictures in each category. A *t*-test further confirmed the significance of this difference (t (160) = 26.76, *p* < 0.001). This study measured and recorded the emotion regulation patterns and EEG activities of college students in response to different social situation pictures.

### 2.3 Procedure

In the experiment, the participants were arranged to sit in front of a 17-inch computer monitor 75 cm away. The experimental programme relied on E-Prime 2.0 software to ensure the standardization and accuracy of the experiment. The display background of the stimulus materials was uniformly set to black to ensure the consistency of visual presentation.

**FIGURE 1:**
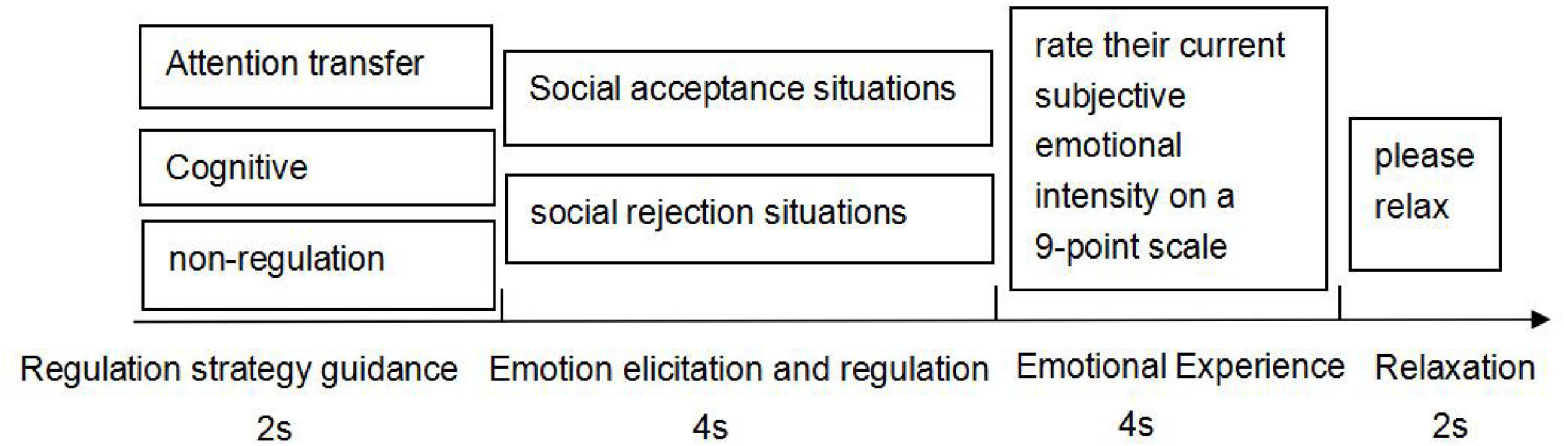
Experimental process chart.

In the experimental process, four consecutive links were carefully designed for each trial: first, one of the three guidelines was randomly displayed on the screen, including “please watch”, “please focus on the observation of camera angle and clarity” and “perform a continuous minus 3 task (from 100)”. The display duration of each guideline was strictly controlled within 2 s. This step aimed to clearly inform the participants of the psychological tasks to be performed next. Next, a “+” fixation point lasting 500 ms appeared at the center of the screen as a hint of visual focus. Then, an emotional picture was randomly presented, covering three types of social acceptance, social rejection and neutral emotions (excluded from experimental conditions), and the display time was 4 s. During this phase, participants performed emotion regulation tasks per instructions: non-regulation (“please watch”), cognitive reappraisal (“please focus on the camera angle/clarity”, operationalized as detached reappraisal via psychological distancing), and attention transfer (“perform serial subtraction”). By establishing objectivity through camera-angle focus (psychological distancing implementation), this cognitive reappraisal promoted cross-context resource homeostasis, thereby reducing contextual heterogeneity in regulatory cost. Participants then proceeded to the emotional intensity assessment phase. They were instructed to rate their current subjective emotional intensity on a 9-point scale displayed on-screen, ranging from 1 (not at all intense) to 9 (extremely intense). This phase lasted for 3 s, and the participants were required to complete their scoring by pressing the key according to their real feelings. Finally, to ensure that the participants could fully relax, the screen displayed the words “please relax” for 2 s as the end sign of the experimental trial (Figure 1). Each participant completed 81 trials per regulation strategy (non-regulation, cognitive reappraisal, attention transfer) × social situation (acceptance, rejection) combination, resulting in 486 experimental trials total. Neutral filler trials (*n* = 54) were interspersed to mitigate habituation but excluded from analysis.

This experiment adopted an in-group experimental design, covering the cross combination of three emotion regulation modes (i.e., non-regulation, attention transfer and cognitive reappraisal) and two social situations (social acceptance and social rejection situations), with neutral stimuli serving exclusively as fillers.

### 2.4 Data collection and analysis

The experiment utilized E-Prime for stimuli presentation and data collection and a 64-channel NeuroScan system for EEG recording. The participants wore a cap with 64 electrodes, and their eye movements were recorded. The left mastoid served as the reference electrode, with a 1000 Hz sampling rate and a 0.01–100 Hz band-pass filter. Scalp impedance was maintained below 5kiloohms, and EEG signals were continuously monitored for data accuracy.

For the collected EEG data, we conducted offline analysis using Scan 4.3 software. During the analysis, the average value of the bilateral mastoids was used as the reference to automatically process the eye movement artifacts and remove the EEG artifacts exceeding ± 100 uV. The time course of EEG analysis was 250 ms before the stimulus and 1500 ms after the stimulus, with the 250 ms before the stimulus serving as the baseline level.

Linear mixed-effects models (LMMs; lme4 package: Bates et al., 2015) were implemented to account for trial-level variability and subject-specific effects inherent to ERP data. Fixed effects included emotion regulation strategy, time window, social context, and their interactions. Random effects encompassed subject-level intercepts, within-subject strategy slopes, and trial-level intercepts. Models were fitted using restricted maximum likelihood (REML) estimation with Satterthwaite-adjusted degrees of freedom (Heise et al., 2022; Volpert-Esmond et al., 2021).

### 2.5 Behavioral data analysis

Herein, the intensity of emotional experience was considered the dependent variable, while the independent variables included situational stimuli (i.e., social rejection and social acceptance) and emotion regulation variables (i.e., non-regulation, cognitive reappraisal and attention transfer). Through ANOVA, the results indicated a significant interaction between emotion regulation and situational stimuli variables, F(2,46) = 3.86, *p* < 0.05, η^2^ = 0.14. Further simple effect tests revealed that in the context of social acceptance, the intensity of emotional experience was significantly higher in the non-regulation mode (*M* = 5.32, SD = 0.26) than in the cognitive reappraisal (*M* = 4.47, SD = 0.28) and attention transfer (*M* = 4.58, SD = 0.29) modes. However, there was no significant difference in emotional experience intensity between cognitive reappraisal and attention transfer. Similarly, in the context of social rejection, the intensity of emotional experience was significantly higher in the non-regulation mode (*M* = 6.31, SD = 0.28) than in the cognitive reappraisal (*M* = 5.15, SD = 0.27) and attention transfer (*M* = 5.46, SD = 0.29) modes. Again, there was no significant difference in emotional experience intensity between cognitive reappraisal and attention transfer.

In addition, the main effect of the emotion regulation variables was significant (F(2,46) = 10.03, *p* < 0.001, η^2^ = 0.31). The intensity of emotional experience in the non-regulation mode (*M* = 5.81, SD = 0.25) was significantly higher than that in the cognitive reappraisal (*M* = 4.82, SD = 0.26) and attention transfer (*M* = 5.01, SD = 0.28) modes. The main effect of contextual stimuli was also significant (F(2,46) = 20.41, *p* < 0.001, η^2^ = 0.47). The intensity of emotional experience in the social rejection context (*M* = 5.64, SD = 0.24) was significantly higher than that in the social acceptance context (*M* = 4.79, SD = 0.25). The behavioral data from this study confirmed the successful activation of social rejection scenarios, and demonstrated that both cognitive reappraisal and attention transfer exhibited effective emotion regulation effects on subjective experience scores.

### 2.6 ERP results

**FIGURE 2:**
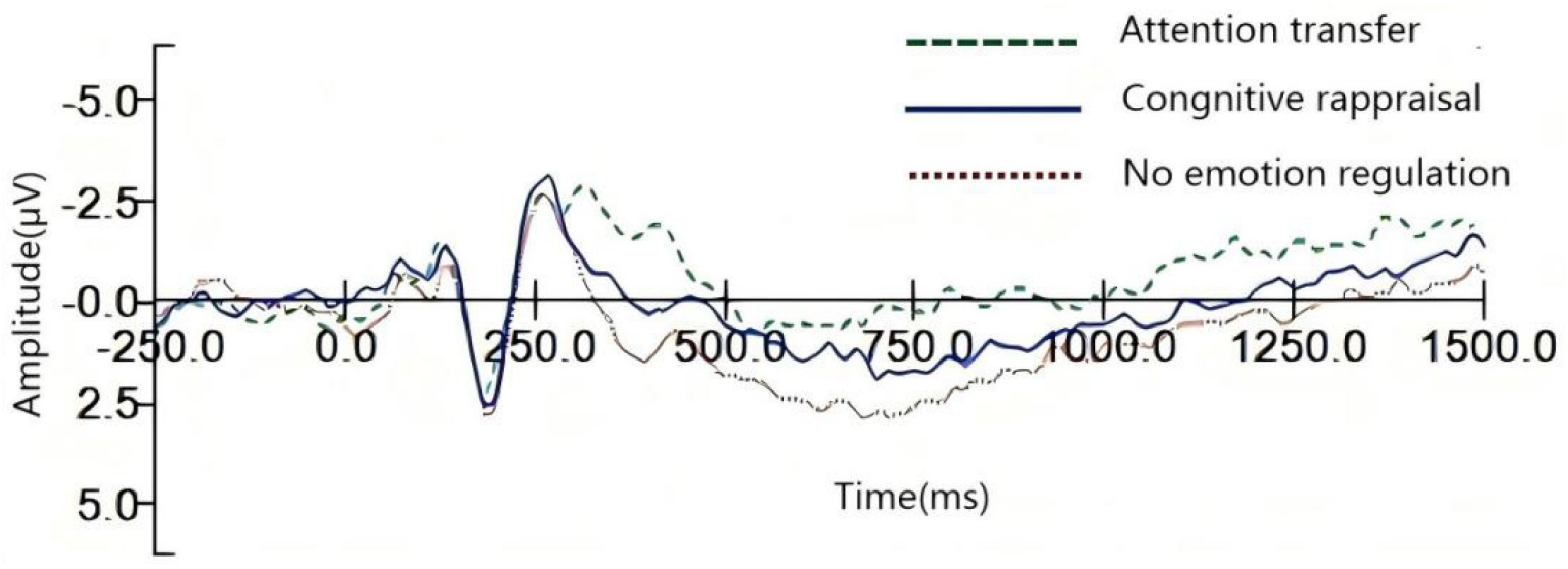
Electroencephalogram of CPz electrode points in the context of social rejection.

Zhang and Lu (2012) established that the CPz electrode site, located over the parietal–occipital region, exhibits amplitude variations sensitive to emotion regulation effects. The present study employed LMMs to examine the impact of distinct emotion regulation strategies–specifically non-regulation, cognitive reappraisal and attention transfer–on LPP amplitude during social rejection. Figure 2 visually depicts the grand-average LPP waveforms elicited during emotion regulation under social rejection. Table 1 displays the mean LPP amplitude values at the CPz site during the regulation epoch across conditions.

**TABLE 1:** Amplitude values of participants at the CPz pole during the emotion regulation stage (*M* ± SD).

	No emotion regulation	Cognitive reappraisal	Attention transfer
LPP (300–600 ms)	3.81 ± 2.65	3.44 ± 2.76	1.74 ± 2.33
LPP (600–1000 ms)	0.75 ± 3.00	0.52 ± 2.91	−1.23 ± 3.06
LPP (1000–1500 ms)	−1.64 ± 2.93	−1.57 ± 2.74	−2.81 ± 3.55

Early Time Window (300–600 ms post-stimulus): A significant main effect of the emotion regulation strategy on LPP amplitude was observed (F(2, 48) = 24.34, *p* < 0.001, η^2^ = 0.50). *Post hoc* comparisons (Bonferroni-corrected) revealed significantly smaller LPP amplitudes under attention transfer compared to both non-regulation (*p* < 0.001) and cognitive reappraisal (*p* < 0.001). No significant difference emerged between non-regulation and cognitive reappraisal (*p* = 0.782).

Mid-Stage Time Window (600–1000 ms post-stimulus): The main effect of the regulation strategy remained significant (F(2, 48) = 18.53, *p* < 0.001, η^2^ = 0.44). *Post hoc* tests confirmed that LPP amplitudes during attention transfer were significantly lower than amplitudes during both non-regulation (*p* < 0.001) and cognitive reappraisal (*p* < 0.001). Amplitudes did not differ significantly between non-regulation and cognitive reappraisal (*p* = 0.421).

Late Time Window (1000–1500 ms post-stimulus): Under social rejection, a significant main effect of the regulation strategy persisted (F(2, 48) = 4.61, *p* = 0.015, η^2^ = 0.16). *Post hoc* analysis indicated that LPP amplitude under attention transfer was significantly smaller than that under both non-regulation (*p* = 0.023) and cognitive reappraisal (*p* = 0.038). No significant difference was found between non-regulation and cognitive reappraisal (*p* = 0.871).

The model included fixed effects for emotion regulation strategy (non-regulation, cognitive reappraisal, attention transfer), time window (300–600 ms, 600–1000 ms, 1000–1500 ms), social context (acceptance, rejection), and their interactions. Random effects comprised subject-level intercepts, strategy slopes within subjects, and trial-level intercepts. Power analysis confirmed adequacy (1-β = 0.93 for LPP effects based on pilot data). The LMM analysis revealed four core findings based on the ERP data:

(1) Main effect of emotion regulation strategy

Strategy significantly modulated LPP amplitudes (β = −1.32 μV, SE = 0.27, *t* = −4.89, *p* < 0.001 for attention transfer vs. non-regulation). Attention transfer elicited amplitudes 1.32 μV lower than non-regulation (95% CI [−1.64, −1.00], *t* = −9.87, *p* < 0.001) and 0.92 μV lower than cognitive reappraisal (95% CI [−1.21, −0.63], *t* = −6.89, *p* < 0.001).

(2) Strategy × Time Window interaction

A significant interaction emerged, F(4, 128.64) = 4.12, *p* = 0.004, η^2^ = 0.03. Simple effects analysis revealed:

- Early window (300–600 ms): Maximal suppression for attention transfer vs. non-regulation (Δ = −3.22 μV, *p* < 0.001), supporting H1 by confirming attention transfer’s early sensory gating effect during acute rejection.
- Mid window (600–1000 ms): Sustained advantage for attention transfer vs cognitive reappraisal (Δ = −1.67 μV, *p* = 0.003), consistent with H2’s prediction of superior resource mobilization via attentional filtering.
- Late window (1000–1500 ms): Maintained suppression for attention transfer vs non-regulation (Δ = −1.43 μV, *p* = 0.021), aligning with H3 as cognitive reappraisal failed to achieve comparable late-stage efficacy.

(3) Strategy × Social Context interaction

Attention transfer showed amplified suppression under social rejection (Δ = −3.42 μV) versus social acceptance (Δ = −2.54 μV), demonstrating context-dependent regulatory optimization under high-threat conditions (Threat-Adaptive Disengagement Model).

(4) Random effects structure

Significant subject-level intercept variance (σ = 0.87) and trial-level fluctuations (σ = 0.54) confirmed the hierarchical nature of ERP data.

**FIGURE 3:**
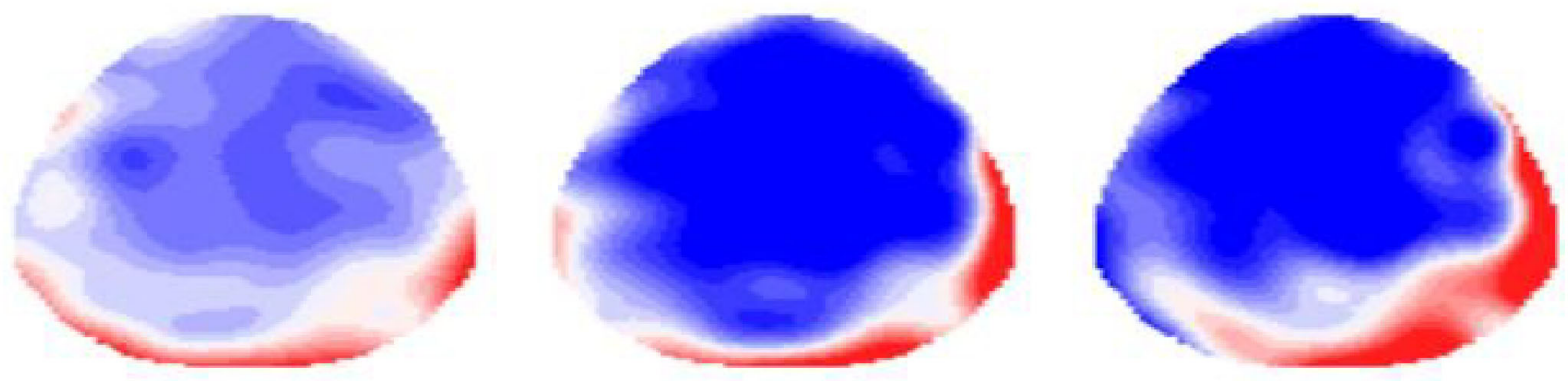
ERP topography of attention transfer effects during social rejection.

Detailed topographical maps (Figure 3) illustrate the effects of emotion regulation, showing changes in the LPP amplitude (i.e., difference amplitude) when individuals employ emotion regulation strategies compared with those who do not in the context of social rejection. A negative actual difference is represented by a blue mark on the EEG topographic map. Further analysis revealed that the topographic map of differential amplitude exhibited distinct dynamic characteristics across different time windows. Initially, when individuals encountered social rejection, their emotion-regulated EEG activity predominantly occurred in the occipital–parietal region, and over time, this activity gradually shifted to the prefrontal region. This process highlights the brain’s dynamic response and adaptive adjustments during emotion regulation.

## 3 Discussion

This study used ERP and subjective measures to examine emotional regulation during social rejection, assess the effectiveness of various strategies and offer cognitive neuroscience insights on individual differences.

### 3.1 Earlier and sustained LPP suppression with attention transfer

Results showed that attention transfer significantly decreased LPP amplitude than cognitive reappraisal and non-regulation. This supports prior research indicating attention transfer effectively lowers LPP amplitude in emotional contexts (Zhang and Lu, 2012). Attention transfer functions during the initial phases of emotional response, effectively suppressing the emergence of emotions by barring emotional stimuli from entering the cognitive processing and evaluation stages (Gross, 2015). Moreover, given that social rejection, as an environment with high-intensity emotional stimuli, is likely to elicit negative emotional experiences in participants, this study utilized a more challenging backward subtraction task. This task necessitated the allocation of substantial attentional resources, prompting participants to shift their focus from emotional stimuli to the task at hand. The strategy exhibited a significant advantage in social situations with strong negative emotions, as it enabled individuals to rapidly disengage from negative emotional information, thereby achieving effective emotional regulation.

Studies have indicated that both cognitive reappraisal and attention transfer can mitigate negative emotions. Contrary to classical reappraisal-LPP modulation patterns (Moser et al., 2009), the operational definition of cognitive reappraisal adopted in this study–which aims to achieve an objective perspective through psychological distancing–likely falls within the domain of detached reappraisal. This strategy exhibited only limited LPP suppression effects during the early stage (300–600 ms). However, prior work reports divergent neural profiles: cognitive reappraisal typically reduces LPP amplitude, while attention transfer paradoxically increases it under certain conditions (Wang et al., 2015). The present study diverges from these findings, demonstrating that attention transfer robustly reduces LPP amplitudes across all stages of social rejection processing. This discrepancy may stem from methodological differences. Wang et al. (2015) utilized repeated exposure to negative stimuli, wherein subsequent attentional deployment might inadvertently trigger defensive hypervigilance–amplifying emotional salience and elevating LPP. Divergence from Wang et al. (2015)–where attention transfer increased LPP–may stem from critical paradigm differences: (1) our use of ecologically valid social rejection stimuli (vs. generic negative images) heightens threat salience, potentiating early gating; (2) serial subtraction’s high working memory load maximized attentional capture, whereas simpler tasks may permit residual emotional processing. By contrast, our paradigm employed ecologically valid social rejection stimuli, allowing attention transfer to function as an early filter that blocks emotional processing before cognitive evaluation (Gross, 2015). Critically, attention transfer achieved significantly lower LPP amplitudes than cognitive reappraisal during both mid-term and late stages. This sustained superiority underscores the effectiveness of attention transfer in high-threat contexts such as social rejection, where rapid disengagement from emotional stimuli is paramount. By engaging working memory to potentially recruit prefrontal regulatory networks (e.g., DLPFC-VLPFC circuits), attention transfer efficiently attenuates limbic reactivity, whereas cognitive reappraisal–reliant on slower prefrontal reconstructive processes–requires prolonged temporal engagement for comparable effects. These spatiotemporal dynamics align with our proposed Threat-Adaptive Disengagement Model, wherein early sensory gating via working memory occupation rapidly mobilizes dorsolateral prefrontal resources to block emotional evaluation circuits under high-threat conditions. ERP topographies suggest anterior shifts consistent with prefrontal involvement, though source localization requires future verification. Despite equivalent subjective efficacy between attention transfer and cognitive reappraisal, their neurophysiological profiles diverged significantly. This dissociation suggests that behavioral measures alone may not capture rapid, resource-efficient disengagement mechanisms. Attention transfer likely achieves affective reduction through early sensory gating that minimizes cortical evaluative processing, whereas cognitive reappraisal requires sustained prefrontal engagement to reconstruct emotional meaning–a process not fully reflected in momentary subjective ratings.

### 3.2 Spatiotemporal dynamics of regulatory neurochronometry

This study conducted a thorough analysis of the differential changes in LPP amplitude resulting from various adjustment methods across different time windows. The LPP amplitude changes induced in college students by social rejection situations followed a distinct temporal progression. In the early time window, emotional processing was primarily focused on four regions of the brain’s posterior area. As time progressed, during the middle time window, emotional processing shifted to the upper and lower regions of the right frontal lobe. In the late time window, emotional processing moved further to four regions at the front of the brain. These findings indicate that when college students experience social rejection, their emotional processing mode generally follows a trajectory of gradual migration from the posterior to the anterior regions of the brain. This conclusion aligns with the outcomes of several previous studies, which suggest that this conclusion aligns with several previous studies indicating that the topography of peak LPP amplitudes progressively shifts from posterior to anterior cortical regions (Gao et al., 2010; Deng et al., 2015; Braunstein et al., 2017). The posterior–anterior LPP shift aligns with predictive coding models of prefrontal error minimization (Friston, 2010; Kanske et al., 2011), reflecting a transfer from stimulus-driven salience detection to top-down control implementation (Hajcak et al., 2020). Critically, this spatiotemporal signature requires intact dorsolateral prefrontal cortex (DLPFC)–ventrolateral prefrontal cortex circuitry to suppress limbic reactivity (Morawetz et al., 2017), explaining its attenuation in anxiety disorders due to prefrontal dysregulation (Price et al., 2021).

This study, utilizing high-temporal-resolution ERP technology, reveals that in high-threat contexts of social rejection, the attention transfer strategy achieves sustained neural efficacy advantages through an early sensory gating mechanism (300–600 ms), as evidenced by significantly lower LPP amplitudes in the early, mid-term and late stages compared to cognitive reappraisal. This superiority stems from the efficient mobilization of working memory resources to engage the DLPFC, thereby blocking emotional stimuli from accessing evaluative cortical pathways (Gross, 2015). LPP suppression under attention transfer may reflect attentional diversion rather than pure affective downregulation. High working memory load during serial subtraction could reduce resources for emotional processing, amplifying LPP attenuation (Hajcak et al., 2020). This contrasts with cognitive reappraisal, where sustained LPP may indicate ongoing evaluative effort despite limited early efficacy. Consistent with our proposed framework, attention transfer achieved rapid LPP suppression during early sensory processing (300–600 ms), supporting H1’s prediction of immediate threat disengagement via working memory gating. This advantage persisted through mid-stage resource mobilization (H2) and late-stage compensatory control (H3), confirming the model’s core tenet: under high-threat social rejection, attentional filtering outperforms cognitive reconstruction in both speed and sustainability.

### 3.3 Clinical implication

Attention transfer demonstrated significantly greater and sustained LPP suppression than cognitive reappraisal across all processing phases during acute social rejection, establishing it as the more effective strategy for attenuating neural responses. This advantage originates from early sensory gating, where working memory mobilization (e.g., serial subtraction) blocks emotional stimuli from evaluative cortices, allowing for persistent limbic suppression. These findings support the Threat-Adaptive Disengagement Model: early attentional filtering outperforms slower prefrontal reconstruction in high-threat contexts. Consequently, clinical interventions for rejection-sensitive disorders should prioritize immediate behavioral tactics (attentional anchoring/sensory refocusing and cognitive occupation tasks) for rapid distress reduction and neurofeedback training leveraging the LPP posterior–anterior shift as a real-time biomarker, thereby aligning interventions with the brain’s regulatory architecture, which include attentional techniques for early threat processing and cognitive restructuring for late stabilization.

### 3.4 Limitations and future directions

The generalizability of our findings is limited by standardized (non-autobiographical) rejection stimuli and a university-exclusive sample, potentially introducing social desirability bias and reduced ecological validity. Future research should: (1) validate dynamics in clinical cohorts with ecological stressors; (2) examine developmental trajectories in emotion regulation neurochronometry; (3) increase sample sizes for power≥0.95; (4) employ multimodal neuroimaging (e.g., fMRI-EEG) to resolve circuit interactions. Although ERP topographic shifts suggest anterior-posterior dynamics (e.g., DLPFC engagement), scalp recordings prevent definitive anatomical localization. Regional inferences (e.g., limbic suppression) thus require source reconstruction (e.g., sLORETA). Trait moderators (e.g., attachment style, trait anxiety) influencing strategy effectiveness were not assessed (Domic-Siede et al., 2025). Computational models indicate attention transfer is preferred under high threat due to rapid implementation (Petter et al., 2025), consistent with our findings. Future work should integrate such frameworks to explain interindividual variability. Critically, behavioral equivalence in subjective ratings does not equate to neural equivalence–divergent pathways can yield similar behavioral outcomes despite distinct neurophysiological profiles. This dissociation likely reflects compensatory mechanisms: whereas attention transfer achieves efficient threat disengagement with minimal metabolic cost, cognitive reappraisal requires delayed prefrontal engagement and significantly greater cognitive effort to attain comparable effects (Morawetz et al., 2017).

## 4 Conclusion

This study provides psychophysiological evidence that attention transfer constitutes the optimal strategy for regulating social rejection. Our findings demonstrate that attention transfer significantly reduces late positive potential (LPP) amplitudes across processing stages, outperforming both cognitive reappraisal and non-regulation conditions. Maximal efficacy emerged during early-to-mid processing stages, enabling rapid disengagement from rejection stimuli. Critically, the observed anterior migration of the LPP peak signals progressive implementation of prefrontal control mechanisms. Collectively, these results establish a mechanistic foundation for clinical interventions targeting rejection sensitivity, advocating integration of attentional strategies with neurofeedback-guided protocols.
